# Supplementary figures and images for: Prospective motion correction improves the sensitivity of fMRI pattern decoding
Source: Hum Brain Mapp. 2018 Jun 8;39(10):4018–31. doi: 10.1002/hbm.24228 (PMC6175330; doi:10.1002/hbm.24228)

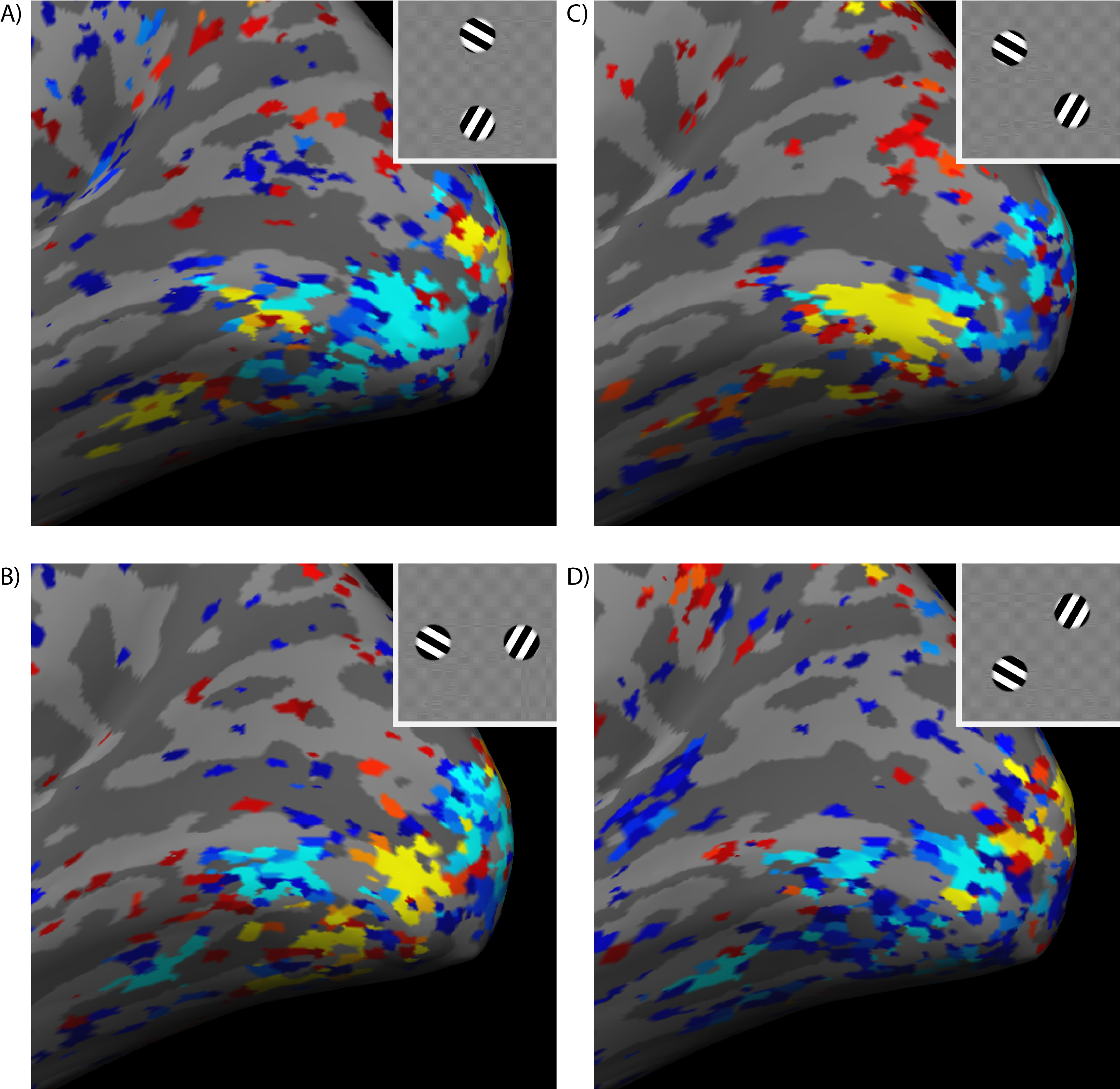

Supplement: Supplementary file 1 — Supporting Information [file HBM-39-4018-s001.tif]

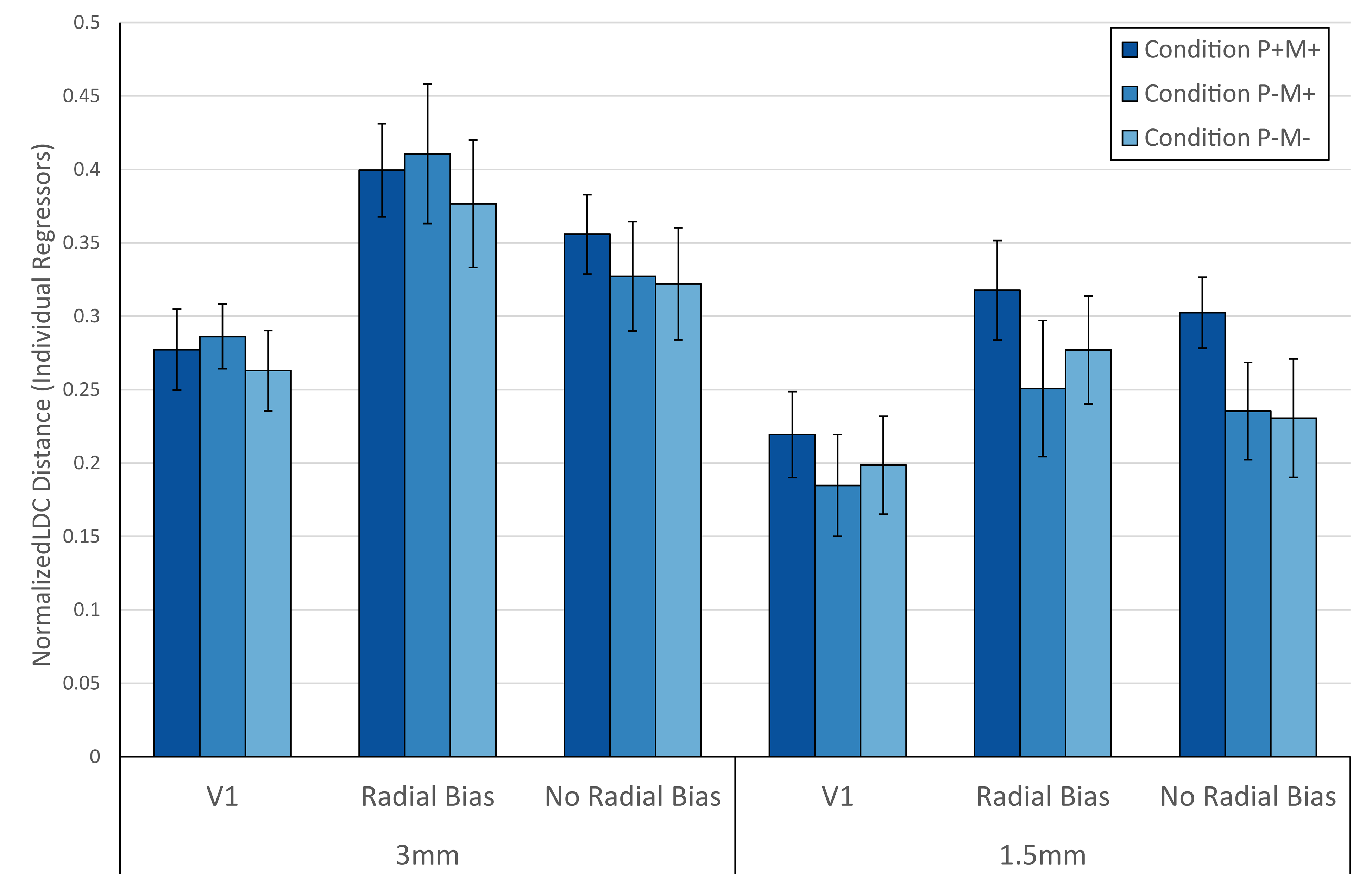

Supplement: Supplementary file 2 — Supporting Information [file HBM-39-4018-s002.tif]

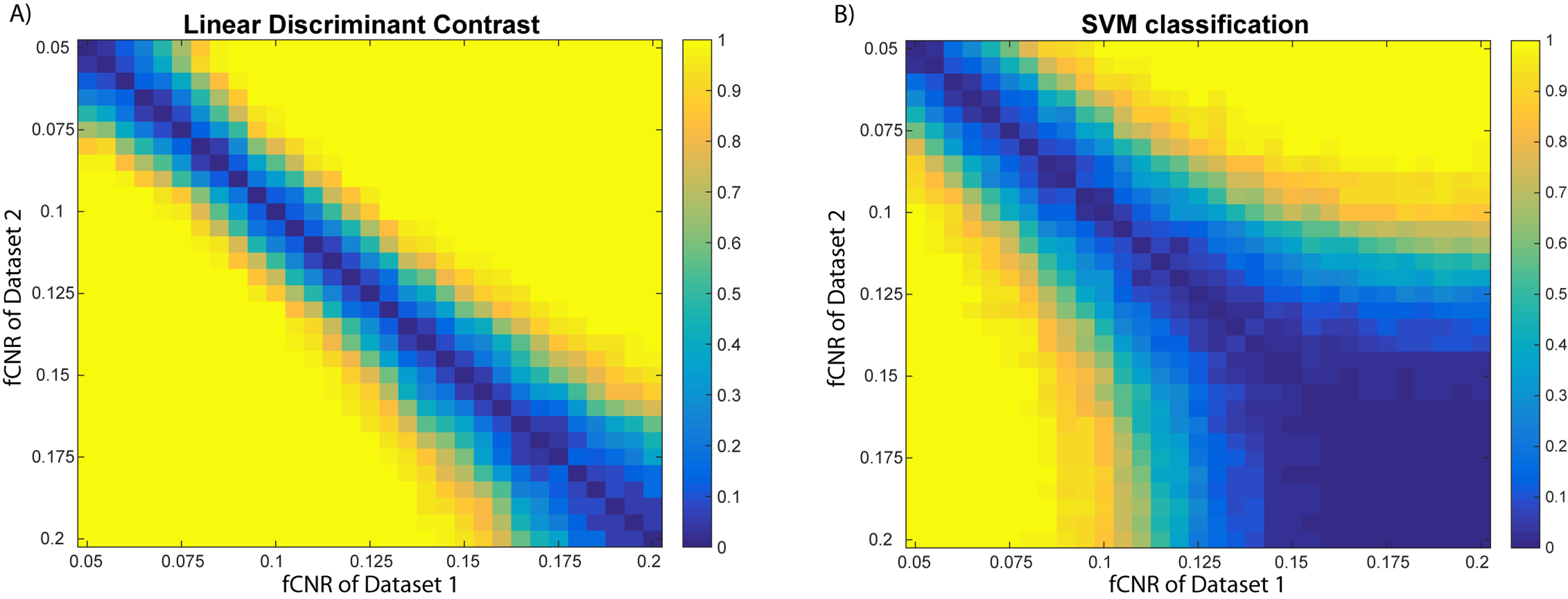

Supplement: Supplementary file 4 — Supporting Information [file HBM-39-4018-s004.tif]

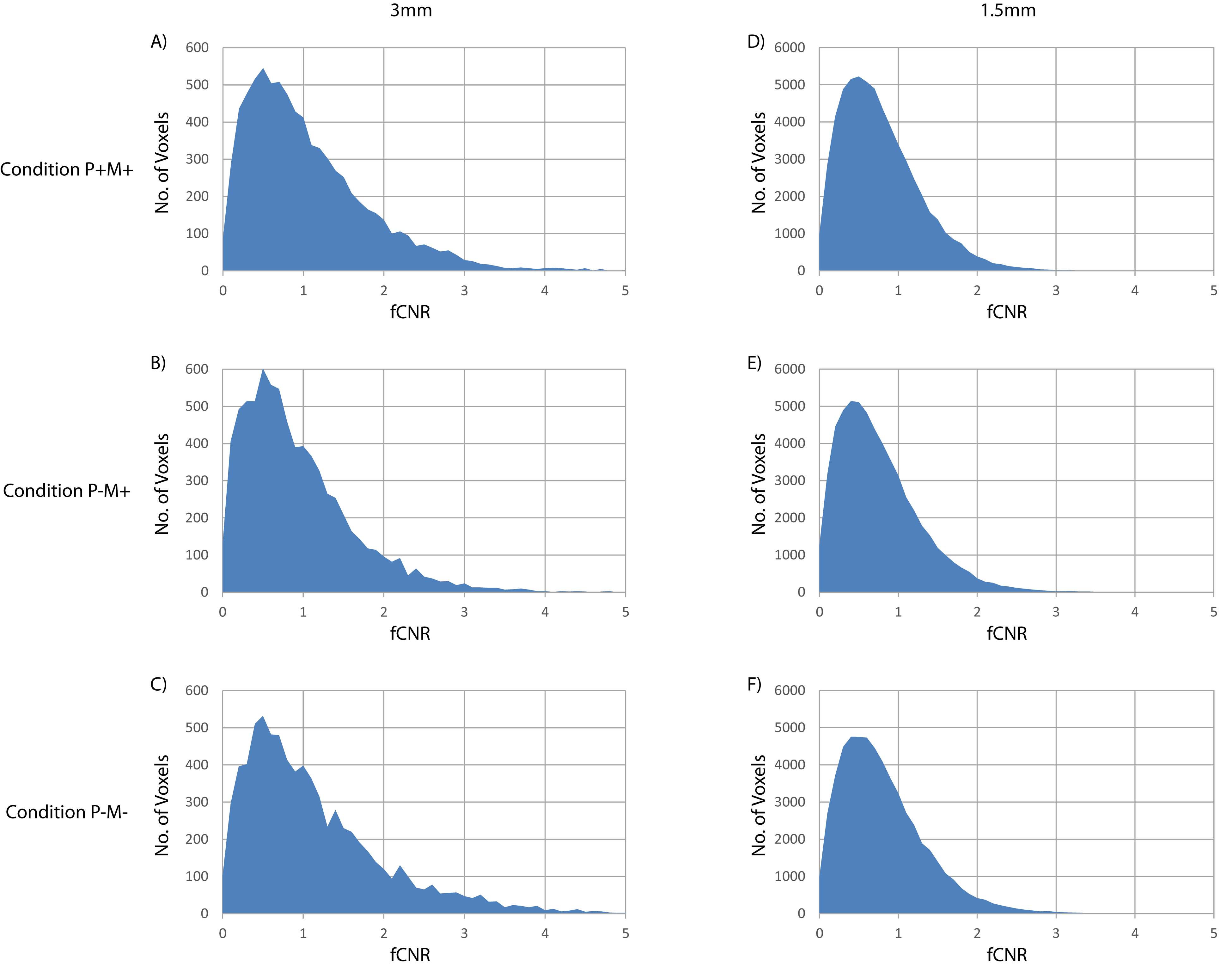

Supplement: Supplementary file 5 — Supporting Information [file HBM-39-4018-s005.tif]
