# Supplementary material for: Prospective motion correction improves the sensitivity of fMRI pattern decoding
Source: Hum Brain Mapp. 2018 Jun 8;39(10):4018–31. doi: 10.1002/hbm.24228 (PMC6175330; doi:10.1002/hbm.24228)
Supplement: Supplementary file 3 — Supporting Information [file HBM-39-4018-s003.docx]

|  | Sum of Squares | DF | Mean Square Error | F | p |
| --- | --- | --- | --- | --- | --- |
| (Intercept) | 22.835 | 1 | 22.835 | 181.79 | 2.07E-09* |
| Error | 1.7585 | 14 | 0.1256 |  |  |
| (Intercept):Resolution | 0.5359 | 1 | 0.5359 | 12.068 | 0.0037* |
| Error(Resolution) | 0.6217 | 14 | 0.0444 |  |  |
| (Intercept):Condition | 0.0615 | 2 | 0.0308 | 0.9063 | 0.4156 |
| Error(Condition) | 0.9502 | 28 | 0.0339 |  |  |
| (Intercept):Region | 0.4578 | 2 | 0.2289 | 10.789 | 0.0003* |
| Error(Region) | 0.5940 | 28 | 0.0212 |  |  |
| (Intercept):Resolution:Condition | 0.0325 | 2 | 0.0163 | 1.0024 | 0.3798 |
| Error(Resolution:Condition) | 0.4540 | 28 | 0.0162 |  |  |
| (Intercept):Resolution:Region | 0.0208 | 2 | 0.0104 | 2.1061 | 0.1406 |
| Error(Resolution:Region) | 0.1383 | 28 | 0.0049 |  |  |
| (Intercept):Condition:Region | 0.0125 | 4 | 0.0031 | 0.7588 | 0.5565 |
| Error(Condition:Region) | 0.2300 | 56 | 0.0041 |  |  |
| (Intercept):Resolution:Condition:Region | 0.0082 | 4 | 0.0020 | 0.6939 | 0.5994 |
| Error(Resolution:Condition:Region) | 0.1647 | 56 | 0.0029 |  |  |

Supplementary Table S3: Repeated measures ANOVA results for LDC distance (individual regressors). * indicates p<0.05 (corrected for multiple comparisons, Tukey’s HSD test). Note that there is a significant effect of resolution, similar to that of classification ANOVA results. This suggests that the improvement of the standard LDC at high resolutions arise due to the simpler model and more stable estimates of the activation patterns.
