# Supplementary material for: Prospective motion correction improves the sensitivity of fMRI pattern decoding
Source: Hum Brain Mapp. 2018 Jun 8;39(10):4018–31. doi: 10.1002/hbm.24228 (PMC6175330; doi:10.1002/hbm.24228)
Supplement: Supplementary file 6 — Supporting Information [file HBM-39-4018-s006.docx]

**Table A**

| ROI 1 | ROI 2 | Difference | Standard Error | p | Lower (95% confidence) | Upper (95% confidence) |
| --- | --- | --- | --- | --- | --- | --- |
| Entire V1 | Radial Bias | -0.1075 | 0.0285 | 0.0055* | -0.1822 | -0.0329 |
| Entire V1 | No Radial Bias | -0.0509 | 0.0212 | 0.0742 | -0.1063 | 0.0046 |
| Radial Bias | No Radial Bias | 0.0567 | 0.0196 | 0.0296* | 0.0055 | 0.1079 |

**Table B**

| Resolution | Condition 1 | Condition 2 | Difference | Standard Error | p | Lower (95% confidence) | Upper (95% confidence) |
| --- | --- | --- | --- | --- | --- | --- | --- |
| 3 | P+M+ | P-M+ | -0.0008 | 0.0272 | 0.9996 | -0.0720 | 0.0705 |
| 3 | P+M+ | P-M- | 0.0230 | 0.0310 | 0.7441 | -0.0582 | 0.1042 |
| 3 | P-M+ | P-M- | 0.0237 | 0.0223 | 0.5496 | -0.0345 | 0.0820 |
| 1.5 | P+M+ | P-M+ | 0.1303 | 0.0369 | 0.0086* | 0.0339 | 0.2268 |
| 1.5 | P+M+ | P-M- | 0.1057 | 0.0530 | 0.1498 | -0.0329 | 0.2443 |
| 1.5 | P-M+ | P-M- | -0.0246 | 0.0452 | 0.8507 | -0.1429 | 0.0937 |

**Table C**

| Reso-lution | ROI | Condition 1 | Condition 2 | Difference | Standard Error | p | Lower (95% confidence) | Upper (95% confidence) |
| --- | --- | --- | --- | --- | --- | --- | --- | --- |
| 3 | Entire V1 | P+M+ | P-M+ | -0.0190 | 0.0249 | 0.7296 | -0.0841 | 0.0461 |
| 3 | Entire V1 | P+M+ | P-M- | 0.0079 | 0.0282 | 0.9583 | -0.0660 | 0.0817 |
| 3 | Entire V1 | P-M+ | P-M- | 0.0269 | 0.0170 | 0.2837 | -0.0175 | 0.0713 |
| 3 | Radial Bias | P+M+ | P-M+ | -0.0146 | 0.0401 | 0.9297 | -0.1196 | 0.0904 |
| 3 | Radial Bias | P+M+ | P-M- | 0.0198 | 0.0444 | 0.8969 | -0.0965 | 0.1361 |
| 3 | Radial Bias | P-M+ | P-M- | 0.0344 | 0.0278 | 0.4503 | -0.0383 | 0.1072 |
| 3 | No Radial Bias | P+M+ | P-M+ | 0.0314 | 0.0309 | 0.5800 | -0.0496 | 0.1124 |
| 3 | No Radial Bias | P+M+ | P-M- | 0.0412 | 0.0301 | 0.3818 | -0.0375 | 0.1200 |
| 3 | No Radial Bias | P-M+ | P-M- | 0.0098 | 0.0282 | 0.9355 | -0.0641 | 0.0838 |
| 1.5 | Entire V1 | P+M+ | P-M+ | 0.1308 | 0.0428 | 0.0219* | 0.0187 | 0.2428 |
| 1.5 | Entire V1 | P+M+ | P-M- | 0.0933 | 0.0587 | 0.2827 | -0.0604 | 0.2469 |
| 1.5 | Entire V1 | P-M+ | P-M- | -0.0375 | 0.0547 | 0.7755 | -0.1806 | 0.1056 |
| 1.5 | Radial Bias | P+M+ | P-M+ | 0.1279 | 0.0480 | 0.0456* | 0.0024 | 0.2535 |
| 1.5 | Radial Bias | P+M+ | P-M- | 0.0808 | 0.0634 | 0.4310 | -0.0850 | 0.2467 |
| 1.5 | Radial Bias | P-M+ | P-M- | -0.0471 | 0.0568 | 0.6920 | -0.1958 | 0.1016 |
| 1.5 | No Radial Bias | P+M+ | P-M+ | 0.1323 | 0.0383 | 0.0101* | 0.0321 | 0.2326 |
| 1.5 | No Radial Bias | P+M+ | P-M- | 0.1430 | 0.0497 | 0.0308* | 0.0129 | 0.2731 |
| 1.5 | No Radial Bias | P-M+ | P-M- | 0.0107 | 0.0375 | 0.9563 | -0.0874 | 0.1088 |

Supplementary Table S6: Multiple Comparison Results for the normalized LDC distance. Table A shows comparisons between ROIs, pooling results across resolutions and conditions. Table B shows comparisons between conditions for each resolution, pooling results across ROIs. Table C shows comparisons between conditions for each resolution and ROI. * indicates p<0.05 (corrected for multiple comparisons, Tukey’s HSD test)
