# Supplementary material for: Prospective motion correction improves the sensitivity of fMRI pattern decoding
Source: Hum Brain Mapp. 2018 Jun 8;39(10):4018–31. doi: 10.1002/hbm.24228 (PMC6175330; doi:10.1002/hbm.24228)
Supplement: Supplementary file 7 — Supporting Information [file HBM-39-4018-s007.docx]

|  | Sum of Squares | DF | Mean Square Error | F | p |
| --- | --- | --- | --- | --- | --- |
| (Intercept) | 1.62E+06 | 1 | 1.62E+06 | 3113.2 | 7.56E-18* |
| Error | 7264.4 | 14 | 518.89 |  |  |
| (Intercept):Resolution | 22975 | 1 | 22975 | 135.3 | 1.39E-08* |
| Error(Resolution) | 2377.2 | 14 | 169.8 |  |  |
| (Intercept):Condition | 1234 | 2 | 617.01 | 1.5679 | 0.2262 |
| Error(Condition) | 11019 | 28 | 393.52 |  |  |
| (Intercept):Region | 243.56 | 2 | 121.78 | 1.5355 | 0.2329 |
| Error(Region) | 2220.6 | 28 | 79.308 |  |  |
| (Intercept):Resolution:Condition | 233.58 | 2 | 116.79 | 0.7888 | 0.4642 |
| Error(Resolution:Condition) | 4145.8 | 28 | 148.06 |  |  |
| (Intercept):Resolution:Region | 323.86 | 2 | 161.93 | 3.0894 | 0.0613 |
| Error(Resolution:Region) | 1467.6 | 28 | 52.414 |  |  |
| (Intercept):Condition:Region | 107.78 | 4 | 26.946 | 0.9255 | 0.4558 |
| Error(Condition:Region) | 1630.5 | 56 | 29.116 |  |  |
| (Intercept):Resolution:Condition:Region | 186.78 | 4 | 46.694 | 2.0529 | 0.0993 |
| Error(Resolution:Condition:Region) | 1273.7 | 56 | 22.745 |  |  |

SUPPLEMENTARY TABLE S7: Repeated measures ANOVA results for classification accuracy. * indicates p<0.05 (corrected for multiple comparisons, Tukey’s HSD test)
